# A Systematic Review of Peer-Support Programs for Smoking Cessation in Disadvantaged Groups

**DOI:** 10.3390/ijerph10115507

**Published:** 2013-10-28

**Authors:** Pauline Ford, Anton Clifford, Kim Gussy, Coral Gartner

**Affiliations:** 1 School of Dentistry, The University of Queensland, 200 Turbot St., Brisbane, QLD 4000, Australia; 2 The Institute for Urban Indigenous Health, 23 Edgar Street, Bowen Hills, QLD 4006, Australia; E-Mails: Anton.Clifford@iuih.org.au (A.C.); Kim.Gussy@iuih.org.au (K.G.); 3 School of Population Health, The University of Queensland, Herston Road, Brisbane, QLD 4006, Australia; 4 University of Queensland Centre for Clinical Research, The University of Queensland, Building 71/918 RBWH Site, Herston, QLD 4029, Australia; E-Mail: c.gartner@uq.edu.au

**Keywords:** peer-support, smoking, cessation, disadvantaged populations

## Abstract

The burden of smoking is borne most by those who are socially disadvantaged and the social gradient in smoking contributes substantially to the health gap between the rich and poor. A number of factors contribute to higher tobacco use among socially disadvantaged populations including social (e.g., low social support for quitting), psychological (e.g., low self-efficacy) and physical factors (e.g., greater nicotine dependence). Current evidence for the effectiveness of peer or partner support interventions in enhancing the success of quit attempts in the general population is equivocal, largely due to study design and lack of a theoretical framework in this research. We conducted a systematic review of peer support interventions for smoking cessation in disadvantaged groups. The eight studies which met the inclusion criteria showed that interventions that improve social support for smoking cessation may be of greater importance to disadvantaged groups who experience fewer opportunities to access such support informally. Peer-support programs are emerging as highly effective and empowering ways for people to manage health issues in a socially supportive context. We discuss the potential for peer-support programs to address the high prevalence of smoking in vulnerable populations and also to build capacity in their communities.

## 1. Introduction

While smoking prevalence in high income countries has fallen substantially in the general population over the past 50 years, the prevalence among disadvantaged sub-populations, such as indigenous peoples, people with severe mental illness and homeless people, within these countries has remained persistently high [1,2,3]. The reasons for the higher smoking prevalence among these sub-populations are likely to be complex and involve multiple factors including social (e.g., high prevalence of smoking among social contacts), psychological (e.g., low self-efficacy) and physical factors (e.g., greater nicotine dependence). These groups have also been subjected to direct targeting by the tobacco industry since at least the 1970s as these “downscale” customers were identified by the industry as an important market [4]. While population level strategies are important to reduce smoking among these groups, effective individual-level strategies are also needed to address the greater barriers faced by people who smoke in these populations [5].

Research shows that unhealthy [6,7] and health promoting behaviours, such as smoking cessation [8] spread through social networks. For example, Christakis and Fowler’s network analysis of the Framingham Heart Study cohort demonstrated that having a social contact quit smoking increased a smoker’s chances of quitting [8]. It is likely that this social contagion effect has enhanced smoking cessation among the general population. By contrast, among sub-populations with a high smoking prevalence, the entrenched smoking culture may have reinforced smoking as the normative behaviour, with non-smokers excluded from social interactions that involve smoking, such as sharing a cigarette [9]. Qualitative research among Australian Indigenous ex-smokers found that supportive relationships were one of the “most useful predictors of successful smoking cessation acting as both a motivator and enabler to behavioural change” [10].

Peer-support programs may be a useful strategy to increase social support for smoking cessation in populations with high smoking prevalence. The generic peer-support model has its roots in the self-help, social justice, human rights and recovery movements [11]. There are many forms of peer-support programs including self-help groups, internet support groups, peer-delivered services, peer-run or operated services, peer partnerships, and peer employees or volunteers within traditional healthcare settings, such as peer companions, peer advocates, consumer case managers, peer specialists, and peer counsellors [12]. Peer-support programs are widely used in the mental health field [13]. They are also becoming important self-management strategies for many chronic conditions, such as diabetes [14,15] and substance addiction [16], and for increasing health-promoting behaviours [17]. Some studies have reported improvements in physical and mental health measures, such as improved glycaemic control, blood pressure, cholesterol, BMI/weight, and depression for participants in peer-support programs [15]. Other benefits of peer-support include positive role-modeling, showing that recovery is possible, and improved socialisation for participants. Peer-support programs can also build capacity among the peer volunteers by increasing their skills, self-efficacy and providing support for maintaining their own abstinence [12,18]. Peers with similar life experiences who have successfully quit may have greater credibility than healthcare staff [12,19]. If so, peer-support may be a highly cost-effective way to provide quitting assistance to people who smoke in populations with a high smoking prevalence. Peer support interventions however have received limited attention in the broader literature on smoking cessation interventions, and were not included in one review of cessation strategies for adults including those in special populations [20].

Four previous reviews of social support and buddy systems in smoking cessation interventions have concluded that there is little rigorous evidence available to support the use of this method [21,22,23,24]. However, three of these reviews included studies targeting general smoker populations in addition to specific sub-populations, including some defined disadvantaged groups. The fourth and more recent of these reviews [24] examined a specific disadvantaged group, adults with severe mental illness. While this review concluded that peer support interventions for this group were promising, the methodology of all but one of the included studies prevented rigorous evaluation of the intervention outcomes. In contrast to these previous reviews, we focus on the evidence from rigorously evaluated studies for peer-support as a smoking cessation intervention in disadvantaged populations only. We hypothesized that peer-support may be more useful in groups where social support may not be readily available or where social networks may act to promote rather than to discourage smoking behaviours.

“Disadvantaged groups” are poorly defined in the literature, although Flaskerud [25] has defined them as “social groups who experience health disparities as a result of lack of resources and/or increased exposure to risk”. For the purposes of this review, the following groups were considered to be defined as disadvantaged: the homeless, prisoners, Indigenous people, those with low socio-economic status (measured by low income/low education or living in a low income area), and people with a mental illness. Smoking during pregnancy is highly associated with low socioeconomic status [26] and therefore interventions targeted at smoking during pregnancy were also included in this definition.

Our aims were to firstly, systematically identify published evaluations of smoking cessation interventions utilizing peer or partner support for disadvantaged groups; secondly, review the key characteristics and outcomes of these programs to determine the extent to which they enhanced the success of quit attempts in these populations; and thirdly, assess their methodological quality.

## 2. Experimental Section

### 2.1. Search Strategy

Electronic databases Embase, Pubmed, CINAHL, Scopus, Web of Science and PsycINFO were searched. The search strategy used was (smoking OR tobacco) AND cessation AND (peer* OR social support* OR social network*). The search was limited to publications in English, studies of humans, publications since 1980 and search terms were required to be in the title/abstract or topic (Web of Science). Search results were further refined using index terms and limits including “tobacco dependence”, “smoking cessation” and “social support”. The search strategy used in PubMed is described in Appendix 1. A total of 1,321 studies were initially identified. In addition, key review articles (*n* = 6) were hand searched for relevant studies, which produced six additional studies. After removal of duplicates this resulted in a total of 1,037 studies.

### 2.2. Selection of Studies

Disadvantaged groups were defined as described above. Peer-support interventions were defined as smoking cessation support delivered by a lay person (*i.e.*, not a health professional or smoking cessation counsellor). Peers could include family members, social or work acquaintances, or volunteers from the target population. Lived experience of smoking was not a prerequisite for inclusion as a peer. If the person providing the support had a formal role in providing health care or social services to the smoker e.g., they were the smoker’s health practitioner or counsellor then the intervention was not defined as being provided by a “peer”. The type of support provided could include cessation advice, general encouragement to quit smoking or accompanying the participant to cessation activities, such as group counselling cessations.

**Figure 1:**
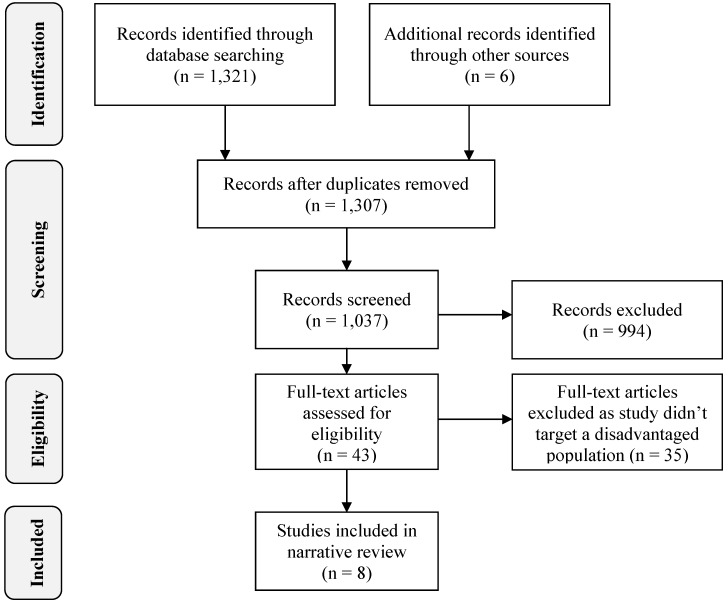
Flow diagram of selection of studies for the systematic review (adapted from [27]).

The abstracts of the 1,037 studies were manually examined by the first author. This initial screening was repeated by two other authors (AC and KG) and any disagreements resolved by discussion by all authors (PF, AC, KG and CG) until consensus was reached. Studies were excluded if they were not original reports of interventions designed to support smoking cessation, did not include a peer support component in the intervention, or did not evaluate the peer support component of the intervention. A total of 994 studies were excluded, leaving 43 studies relevant to this review. The full-text articles of the 43 relevant studies were independently examined by two of the authors (PF and AC) and only those primarily targeting/recruiting disadvantaged populations were included. Interventions targeting the general population or a specific population not defined as disadvantaged were excluded. Where there was disagreement (*N* = 2), a third author (CG) reviewed the study to determine eligibility. A final list of eight relevant studies was reached through consensus (Figure 1).

### 2.3. Review Format and Criteria

Criteria for data extraction from studies were adapted from the Cochrane Collaboration’s Handbook: Systematic Reviews of Health Promotion and Public Health Interventions [28]. The criteria relate to the intervention/s sample (including eligibility, size, age range, and percent male), outcomes measured, and intervention effectiveness. Due to the heterogeneity between the studies in terms of the interventions, populations and outcome measures, we performed a narrative review rather than a meta-analysis. The study population, intervention, evaluation methods and outcomes, and the quality measure for each study are presented in Table 1. A summary score for the effectiveness of the intervention was created: 0 = no effect; 1 = short term effect (less than 3 months); 2 = mid-term effect (3–6 months); 3 = long-term effect (more than 6 months). Abstinence was the outcome measure used for assessment of effectiveness, and non-significant results, even if a trend was demonstrated, were treated as having no effect.

The methodological quality of studies was assessed using the Dictionary for the Effective Public Health Practice Project Quality Assessment Tool for Quantitative Studies [28]. Sections A to F (A = selection bias; B = allocation bias; C = confounders; D = blinding; E = data collection methods; and F = withdrawal and drop-outs) were coded weak, moderate, or strong, consistent with the component rating scale of the dictionary [28]. For Sections G (analysis) and H (intervention integrity), descriptive information was recorded, using dictionary recommendations as a guide. In order to assess the likelihood of publication bias, log odds ratios for each study (where they could be calculated) were plotted against sample size.

**Table 1:** Characteristics of the included evaluations of peer support smoking interventions.

Author, Year, Country	Study Population				Intervention						
	Description of smokers	n	Mean or Median Age (year)	Baseline smoking	Duration	Design	Peers/Partners	Intervention integrity	Length of follow up (% followed-up)	Behaviour change	Summary Efficacy Score
Albrecht *et al*. 2006, USA [29]	Pregnant adolescents (14–19y) from low socio-economic areas; 53% Caucasian; 42% African American; 5% other	142	17	UC = 6.76cpd TFS = 7.04cpd TFSB = 7.31cpd	8 weeks	Usual Care (UC) = 45–60 min individual education session and written materials Teen Fresh Start (TFS) = 8 group sessions Teen Fresh Start plus Buddy (TFSB) = TFS + participants required to identify and bring a peer supporter to sessions	Peer supporters were non-smoking females of similar age identified by the participants. No peer training.	Nurses certified in intervention delivery Participant’s attendance at meeting recorded. Intervention exposure not clearly reported.	1year (53%)	Abstinence at 8wks: TFSB *vs*. UC (*p* = 0.01); no differences at 1year. Low power reported. OR(8 wks) = 3.730	1
Hennrikus *et al*. 2010, USA [30]	Low income pregnant women who smoked aged 18+; 67% racial minority/Hispanic; 65% had a high school education or less 48% married/de facto	82	24	Median = 5cpd; 52% smoked first cigarette within 30 min of waking	Variable depending on due date (approx. 6 months)	Participants identified a woman in their social network to help them quit. Dyads were then randomized to intervention or control groups. Intervention: supporters received monthly contacts from counselor Control: supporters not contacted	Supporter session discussed activities to support participant’s quit efforts; monthly calls reviewed support efforts and planned for next month 52% of supporters were current smokers, 22% were former smokers	Participant attendance recorded Intervention exposure > 89%	3 months pp (68%)	Abstinence at birth: intervention 13.0%; control 3.6%. Abstinence at 3 months pp: intervention 9.3%; control 0%. No statistically significant differences. Participants with friends as supporters more likely to quit (21.7%) than with relatives (6.5%); and more quits when supporters were ex-smokers (18.2%) than never (13.3%) or current (10.7%) smokers. Low power reported. OR unable to be calculated due to small numbers	0
McBride *et al*. 2004, USA [31]	Pregnant women who smoked and recent quitters at an army medical centre living with a partner; 77% Caucasian; 50% employed; 52% more than high school education	583	24	Mean = 13cpd; 33% smoked first cigarette within 30 min of waking	Variable depending on due date (approx. 10 months)	Usual Care (UC): advice at prenatal visit to quit smoking + self-help guide; Woman Only (WO): UC + late pregnancy relapse prevention kit and six counseling calls completed by 4 moths postpartum; Partner Assisted (PA): WO + partner adjunct in which partner advised how to be a quit coach.	Partner training covered helpful/unhelpful behaviours, partners also given assistance to quit if they smoked.	Intervention exposure = Number of counselling calls reported Self-report of partner interaction by woman and support partner Intervention exposure not clearly reported.	1year pp (75%)	Abstinence at 28 weeks of pregnancy UC 60%, WO 59%, PA 61%; Abstinence at 2 months pp UC 38%, WO 37%, PA 42%; Abstinence at 6 months pp UC 33%, WO 36%, PA 37%; Abstinence at 12 months pp UC 29%, WO 32%, PA 35%; Sustained abstinence: UC 15%, WO 20%, PA 21%. No statistically significant differences. No power analysis reported. OR(2 months pp) = 1.186	0
Solomon *et al*. 2000a, USA [32]	Pregnant women, mostly Caucasian, English speaking, low income, low education	151	23.5	Mean = 10.5cpd (intervention); 9.8cpd (control)	Variable depending on due date (approx. 6 months)	Control: brief advice at first 3 pre-natal visits + printed materials. Intervention: Control + offer of telephone peer support for women with moderate or high intentions to quit during pregnancy	Peer supporter (woman ex-smoker) received 8h training	Number and duration of support calls recorded Quality control checks conducted on women in intervention group Intervention exposure > 80%	End of pregnancy (approx. 6 months) (73%)	Abstinence at end of pregnancy: intervention 19%; control 17%. No statistically significant differences. Low power reported. OR(pp) = 1.273	0
Solomon *et al*. 2000b, USA [33]	Low income women	214	33	Mean = 23.7cpd	3 months	Control: free nicotine patches Intervention: free nicotine patches + pro-active telephone peer support	Peer supporter (woman ex-smoker) received 7 h training	Phone support personnel trained Intervention exposure = 53%	6 months (90%)	Abstinence at 3 months: intervention 42%; control 28% (*p* = 0.03). At 6 months, no significant difference. No power analysis reported. OR(3 months) = 1.845	2
Solomon *et al.* 2005, USA [34]	Low income women	330	33.7 (intervention) 34.8 (control)	Mean = 23.6cpd	4 months	Control: free nicotine patches Intervention: free nicotine patches + pro-active telephone peer support	Peer supporter (woman ex-smoker) received 8h training	Number and duration of support calls recorded Intervention exposure~70%	6 months (87%)	Abstinence at 3 months: intervention 42.7%; control 26.4% (*p* = 0.002). At 6 months, no significant difference. Power = 0.40. OR(3 months) = 2.075	2
West *et al*. 1998, UK [35]	Economically and socially disadvantaged	172	42.6 (intervention) 44.5 (control)	FTND = 4.9 (intervention); 5.1 (control)	5 weeks	Control: brief intervention + NRT Intervention: brief intervention + NRT + buddy (paired with another smoker participant)	No peer training (participants were paired with each other)	Level of buddy interaction and use of pharmacotherapy self-reported Intervention exposure = 85%	5 weeks (nr)	Abstinence at end of intervention: intervention 27%; control 12% (*p* < 0.01). Low power reported. OR(5 weeks) = 2.794	1
Williams *et al*. 2011 [36]	People with mental illness (outpatients)	102	43.5	Mean = 19cpd	One off 20 min brief intervention	Pre post study design. Intervention: 20min brief intervention with peer counsellor	Peer counselors are mental health consumers with a min 1year tobacco-free period who receive 30 h intensive training and a detailed training manual.	Weekly phone and face to face supervision and feedback to peer counsellours. Monitoring of number of visits, events and smokers receiving intervention. Intervention exposure = 100%	6 months (59%)	Reduction in cpd at 1 month (*p* < 0.001) compared with baselineReduction in cpd at 6 months (*p* = 0.001) compared with baseline	2

nr = not reported.

## 3. Results and Discussion

All but one included studies were randomized controlled trials.

### 3.1. Study Populations

The included studies reported interventions for a range of disadvantaged groups. Half (four studies) targeted pregnant women of low socioeconomic status [29,30,31,32]. Two studies targeted women from low socioeconomic populations [33,34], and one study was designed for a general population from an economically and socially disadvantaged area [35]. One study reported an intervention for people with mental illness [36]. Of note, none of the included studies reported interventions for Indigenous populations, homeless people, or prisoners, even though these are some of the populations with the highest smoking prevalence.

### 3.2. Efficacy

Summary scores (Table 1) showed that three of the seven studies demonstrated no significant effect of the peer-support intervention on abstinence [30,31,32]. Two studies reported a short-term effect [29,35], and three studies showed a mid-term effect [33,34,36]. No studies reported an effect that lasted longer than 6 months. One of the studies that demonstrated no effect was an intervention to determine whether training of peer-supporters increased the likelihood of participants quitting [30]. All participants were asked to identify another woman from their social network to provide support, but only in the intervention group did the peer-supporters receive training. It is possible that this study design was not adequate to detect an effect above that observed by having an untrained peer-supporter. In most studies, control group interventions were often quite substantial and it may be that any improvement in quit rates by the addition of peer-support to the intervention was not of a large enough magnitude to demonstrate statistical significance. A likely explanation for the lack of observed statistically significant differences between intervention and control groups in a number of studies is that the studies were inadequately powered. It is interesting to note that only one of the four studies targeting pregnant women who smoke demonstrated an effect, and this was only at the 8 week follow up, which occurred during pregnancy [29]. There is a possibility that once the birth had occurred, motivation reduced and stress increased for participants, making relapse more likely. This suggests that pregnant women who smoke represent a particularly challenging group for smoking cessation interventions. The remaining studies [33,34,35,36] (not targeting pregnant women) all demonstrated some significant effect of peer-support on abstinence or reduced cigarette smoking, albeit at short- and mid-term follow ups only. Two of these studies showed a significant effect at three-months, but this effect was no longer present by the six month follow-up, which occurred after the peer-support had ended two or three months previously [33,34]. The intervention targeting people with mental illness demonstrated a significant reduction in cigarettes per day at both the one month and six month follow ups [36]. Motivation to quit once a smoker becomes pregnant is increased and this has been described as a “window of opportunity” for smoking cessation [37]. Quit rates are very high during pregnancy, whether quitting occurs spontaneously or as a result of an intervention. Unfortunately, however, relapse in the postpartum period occurs for the majority of quitters, with evidence suggesting that extending interventions into the postpartum period merely delays rather than prevents relapse. Smoking during pregnancy is associated with socioeconomic disadvantage and co-habiting with a smoker [26]. To more effectively address smoking for this group, broader factors such as life stressors and partner support may also need to be targeted [37].

### 3.3. Intervention Design

Most interventions consisted of a package of support measures such as NRT, information and behavioural skills training. This complexity meant that a rigorous study design, often with multiple arms, was required to enable separate evaluation of the peer-support component. Several studies were excluded from the review due to the study design limitations which did not allow the peer-support component to be evaluated. The nature of the buddy/support person relationship meant that there were usually multiple contacts/interactions throughout the intervention period compared with a lesser number of contacts for control group participants, and this is likely to have been one of the factors explaining observed efficacy. Interventions varied in duration but were generally conducted over several months.

### 3.4. Peer Selection and Training

For three of the included studies, participants were asked to identify a peer from their social network or their partner as their support person for the intervention [29,30,31]. In two of those studies, the peer/partner received training on how to support the participant in their quit attempt, however neither of these studies demonstrated improved quit rates by the peer-support intervention [30,31]. In one study, participants (by definition smokers) were paired with each other to form the peer-support [35]. In three studies, female ex-smokers were trained to provide telephone support to the participants [32,33,34]. The three studies that demonstrated a mid-term effect were those where the peer-support was provided by peers who had received more extended and structured training [33,34,36]. Two of these interventions also included provision of free NRT, and so it is possible that one of the advantages of the trained peer was that advice on how to use NRT effectively was provided [33,34]. For the remaining studies, it was unclear whether there were benefits in training the peers or whether the smoking status of the peer influenced the outcomes. A potential benefit of training peers from the participant’s own network is that support could conceivably continue longer term, that is beyond the duration of the intervention. Training may also have broader benefits such as the development of positive relationship skills and the ability to support other people who smoke in their social network.

### 3.5. Theoretical Frameworks

As identified in a recent review [22], interventions using peer-support to enhance smoking cessation generally demonstrate little reference to the theoretical underpinnings of social support. In evaluating these interventions, measurement of potential mediators of the desired outcome (abstinence) should be included. It is important to know whether the intervention was successful in enhancing peer-support in order to interpret the observed abstinence rates. Only three of the eight studies included some measurement of social support constructs [29,30,31]. Only one of these studies reported an enhancement of social support by the intervention [30]. It seems clear that if the intervention is not successful in providing helpful support for the smoker in their quit attempt, then it cannot be expected to lead to enhanced abstinence. Understanding the extent to which peer-support interventions are successful in enhancing social support to the smoker is therefore critical to further research. One study [31] utilized the Partner Interaction Questionnaire (PIQ) [38], which is commonly used to evaluate peer-support. This instrument assesses positive and negative perceived and provided support for cessation.

### 3.6. Methodological Quality

Most studies were rated strong on methodological quality criteria related to study design, data collection and analyses, and confounders. Seven of the eight study designs employed randomisation and a control group thereby reducing the risk of allocation bias [29,30,31,32,33,34,35]. One study employed a pre-post design [36]. Outcome data for seven of the eight studies was collected using reliable and valid measurement instruments and analysed using an intent-to-treat analysis [29,30,31,32,33,34,35]. Six of the eight studies were rated as low risk of confounding on the basis that they reported there were no important differences between groups prior to the intervention or they controlled for differences in the analysis [29,31,32,33,34,35]. Of the other two studies, one was rated moderate for confounding because important differences between groups were identified but not adequately controlled for in the analysis [30], and the other weak because its non-randomised design reduced the ability to sufficiently control for confounding variables [36].

Ratings for methodological quality criteria related to selection bias and withdrawal and drop-outs were variable across studies. Four studies were rated moderate [29,32,33,35] and four weak [30,31,34,36] for selection bias. Two studies reported >80% of eligible individuals agreed to participate [30,34] but were rated moderate for selection bias as participants were not randomly selected from the target population. The four studies rated weak for selection bias reported <60% of eligible individuals agreed to participate [30,31,34,36]. The variability in ratings across studies for withdrawal and drop-out criterion was largely due to differences in follow-up rates: two studies reported follow-up rates > 80% [33,34]; five reported follow-up rates ranging from 53% to 75% [29,30,31,32,36]; and one did not clearly report follow-up rates [35].

No study performed a cost analysis and none reported blinding. However, the nature of the intervention would make effective blinding difficult. Economic analysis is important for understanding resources and the potential cost-effectiveness of peer-support strategies designed to enhance smoking cessation and subsequent economic cost and social savings [39]. The evidence base for the cost-effectiveness of peer-support interventions for smoking cessation in disadvantaged groups would be strengthened by evaluation studies that recruit more representative samples, improve consent and follow-up rates, and conduct high-quality economic evaluations.

While the evidence is mixed, research in general populations suggests that peer-support smoking cessation programs can assist quitting. Two studies employing dyad peer-based interventions to promote smoking cessation reported greater quitting among those receiving peer-support than controls (ORs were 1.3 and 1.8) [17]. May and West’s review of “buddy systems” for smoking cessation concluded that the addition of buddy systems to smoking cessation clinic support may benefit quitters [21].

None of the studies included in this review targeted Indigenous or First Nations populations, such as Aboriginal and Torres Strait Islanders, New Zealand Maori, American Indian, or Alaska Native peoples. Peer-support programs may be particularly suitable for supporting behaviour change among Indigenous people as peer-support programs have a strong emphasis on social empowerment and align with cultural approaches and values such as the mentoring role of Elders [40]. However, there has been little research on the effectiveness of peer-support programs for smoking cessation among Indigenous populations [5]. A cross-sectional survey of peer-support preferences among urban-dwelling Indigenous people in Melbourne, Australia, found that of the smoking participants, half would prefer to receive support to quit smoking in the form of a weekly group meeting, a third would prefer face-to-face counselling, while only 20% and 10% reported interest in receiving support in the form of website/emails and phone counselling, respectively [41]. Approximately one quarter of the respondents were interested in being trained to be a volunteer peer-mentor [41]. These researchers then developed a peer-mentoring program to address multiple behaviours (physical activity, fruit and vegetable consumption and smoking cessation) [18]. In the development of this program, the potential volunteer peers expressed a preference for an informal program that utilised their existing social connections with the local Indigenous community. Unfortunately, while the authors report that some participants in the program quit smoking, limited information was provided on the activities of the peers and whether participants were referred to and accessed formal smoking cessation support in their community. Further research is needed on whether peer-support programs are effective strategies to increase quit attempts and the success of these attempts among Aboriginal and Torres Strait Islander people who smoke and other Indigenous and First Nations populations.

**Figure 2:**
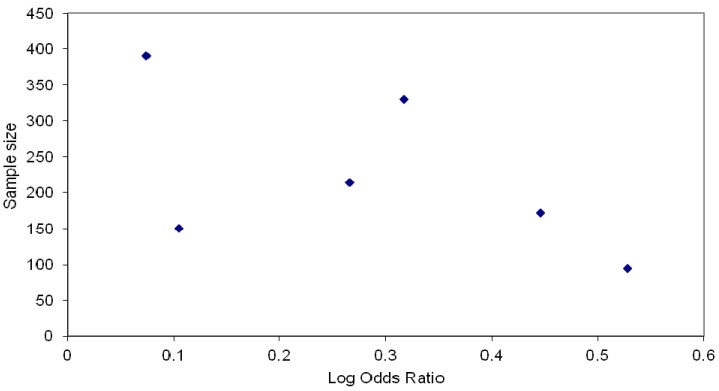
Funnel Plot.

### 3.7. Publication Bias

Odds ratios were extracted from the data reported for each study for short term follow up (no longer than 3 months), apart from two studies [30,36] where small numbers or study design prevented calculation (Table 1). Figure 2 demonstrates that the effect size followed a symmetrical distribution with respect to the sample size, with the exception being one study which had the largest sample size but the smallest effect. While these results should be interpreted with caution due to the variation in intervention types, follow up times and control group interventions, there is no evidence for publication bias.

## 4. Conclusions

This review has demonstrated some, albeit limited evidence for the efficacy of peer-support in smoking cessation for disadvantaged groups. It has also highlighted that there are substantial gaps in this evidence base. There were only a small number of studies identified with designs that allowed separate evaluation of the peer-support component, indicating that there are challenges in implementing RCTs with adequate statistical power, and acceptable levels of attrition and loss to follow up in these population groups. Importantly, there was only one included study which targeted a highly disadvantaged group, those with mental illness. There were no included studies targeting Indigenous, migrant, refugee, incarcerated or homeless populations.

In contrast with previous reviews of this intervention type in the general smoker population, our study has suggested more promising results when peer-support is implemented as a smoking cessation method in economically and socially disadvantaged populations. While short- and mid-term improvements in abstinence appeared achievable, more work needs to be done on improving the sustainability of the peer-support beyond the formal intervention if longer-term outcomes are to be achieved. Capacity building by training peers from the smoker’s own social network seems worthy of further investigation. Results for disadvantaged pregnant women who smoke were less clear, indicating that the event of birth may introduce additional complexities and challenges to quit attempts in these groups. Further research is needed to provide more rigorous evidence regarding the most cost-effective interventions for smoking cessation in disadvantaged groups.

## Appendix 1

PubMed search strategy 1980 to February 2013 (limits: humans, English)

1. smoking.ti,ab
2. tobacco.ti,ab.
3. 1 OR 2
4. cessation.ti,ab
5. peer*.ti,ab.
6. social support*.ti,ab.
7. social network*.ti,ab.
8. 5 OR 6 OR 7
9. 3 AND 4 AND 8
10. smoking cessation/
11. social support/
12. 9 AND 10 AND 11
